# Development and deployment of KASP markers for multiple alleles of *Lr34* in wheat

**DOI:** 10.1007/s00122-020-03589-x

**Published:** 2020-04-12

**Authors:** Tilin Fang, Lei Lei, Genqiao Li, Carol Powers, Robert M. Hunger, Brett F. Carver, Liuling Yan

**Affiliations:** 1 Department of Plant and Soil Sciences Department, Oklahoma State University, 368 AG Hall, Stillwater, OK 74078 USA; 2 Entomology and Plant Pathology Department, Oklahoma State University, Stillwater, OK 74078 USA

## Abstract

**Key message:**

Heterogeneous Lr34 genes for leaf rust in winter wheat cultivar ‘Duster’ and KASP markers for allelic variation in exon 11 and exon 22 of Lr34.

**Abstract:**

Wheat, *Triticum aestivum* (2*n* = 6*x* = 42, AABBDD), is a hexaploid species, and each of three homoeologous genomes A, B, and D should have one copy for a gene in its ancestral form if the gene has no duplication. Previously reported leaf rust resistance gene *Lr34* has one copy on the short arm of chromosome 7D in hexaploid wheat, and allelic variation in *Lr34* is in intron 4, exon 11, exon 12, or exon 22. In this study, we discovered that Oklahoma hard red winter wheat cultivar ‘Duster’ (PI 644,016) has two copies of the *Lr34* gene, the resistance allele *Lr34a* and the susceptibility allele *Lr34b*. Both *Lr34a* and *Lr34b* were mapped in the same linkage group on chromosome 7D in a doubled-haploid population generated from a cross between Duster and a winter wheat cultivar ‘Billings’ which carries the susceptibility allele *Lr34c*. A chromosomal fragment including *Lr34* and at least two neighboring genes on its proximal side but excluding genes on its distal side was duplicated in Duster. The Duster *Lr34ab* allele was associated with tip necrosis and increased resistance against leaf rust at adult plants in the Duster × Billings DH population tested in the field, demonstrating the function of the Duster *Lr34ab* allele in wheat. We have developed KASP markers for allelic variation in exon 11 and exon 22 of *Lr34* in wheat. These markers can be utilized to accelerate the selection of *Lr34* in wheat.

## Introduction

Wheat, *Triticum aestivum* (2*n* = 6*x* = 42, AABBDD), is an allopolyploid produced from two separate hybridization events. The first hybridization event occurred between the two diploid grass species *T. urartu* (the A genome donor, 2*n* = 2*x* = 14, AA) and *Aegilops speltoides* (the possible B genome donor, 2*n* = 2*x* = 14, BB) to form the tetraploid *T. turgidum* (2*n* = 4*x* = 28, AABB), and the second hybridization event occurred between the tetraploid species and a diploid goatgrass species *Ae. tauschii* (the D genome donor, 2*n* = 2*x* = 14, DD) to form the hexaploid *T. aestivum* (McFadden and Sears 1946; Dvořák et al. 1993; Wang et al. 1997; Huang et al. 2002; Chalupska et al. 2008). Each of the three homoeologous genomes A, B, and D should have one copy for a gene in its ancestral form if the gene has no duplication or deletion. The leaf rust resistance gene *Lr34* is a simpler name for the *Lr34/Yr18/Sr57/Pm38* genes on the short arm of chromosome 7D in hexaploid wheat that confer non-specific resistance against fungal pathogens including leaf rust, stripe rust, stem rust, and powdery mildew, as well as barley yellow dwarf virus (Krattinger et al. 2009, 2013; Lagudah et al. 2009; Risk et al. 2013). Because of the durability and non-specificity of its resistance against multiple pathogens, *Lr34* has become one of the most important disease-resistance genes in wheat worldwide. Wheat breeding programs have capitalized on naturally occurring but widespread variation for this gene, as it remains a prioritized target where fungal disease protection is critical (Spielmeyer et al. 2013; Ellis et al. 2014).

Current understanding on the *Lr34* gene in hexaploid wheat is that only one copy exists on each of the three homoeologous genomes. *Lr34* on the short arm of chromosome 7D, which hereafter is referred to as *Lr34*. Only a single copy of *Lr34* is detected in the genome of diploid wheat species *Ae. tauschii*, and its homologue is found in other diploid grass species such as rice (*Oryza sativa*) and sorghum (*Sorghum bicolor*) but not in maize (*Zea mays*), barley (*Hordeum vulgare*), and Brachypodium (Krattinger et al. 2013). The *Lr34*gene is unique to the wheat D genome, and its orthologue in the A or B genome of hexaploid wheat lost function. However, the resistance of *Lr34* against different diseases was detectable when it was over-expressed in cereal crops including rice (Krattinger et al. 2016), Sorghum (Schnippenkoetter et al. 2017), maize (Sucher et al. 2017), barley (Boni et al. 2018), as well as tetraploid durum wheat containing A and B genomes (Rinaldo et al. 2017).

Seven alleles, *Lr34a* through *Lr34g*, have been identified for *Lr34*, and the first resistance allele in cultivars such as ‘Chinese Spring’ is hereafter is referred to as *Lr34a* (Lagudah 2011). Current understanding of allelic variation in *Lr34* is that *Lr34a* gained function from natural mutations of *Lr34b* for the first susceptibility allele as an ancestral form in cultivars such as ‘Renan’ (Krattinger et al. 2009; Lagudah et al. 2009; Lagudah 2011). The *Lr34* allele consists of 24 exons and 23 introns spanning 11,805-bp nucleotide sequence from the start codon to the stop codon for translation and encodes a pleiotropic drug resistance (PDR)-like adenosine triphosphate-binding cassette (ABC) transporter (Krattinger et al. 2009). Although additional resistance or susceptibility alleles exist in genetically divergent wheat germplasm (Dakouri et al. 2010), the *Lr34* gene region has been well characterized for functional differences, such that *Lr34a* and *Lr34b* can be distinguished by three polymorphisms. The first one is a single-nucleotide polymorphism (SNP) in intron 4, the second one is a codon ‘TTC’ encoding phenylalanine present in exon 11 in the susceptibility allele but absent in the resistance allele, and the third one is in exon 12, which is involved in encoding a tyrosine in the Lr34b protein but a histidine in the Lr34a protein. The gain-of-function model of *Lr34* from the TTC-C haplotype for the *Lr34b* allele to the XXX-T haplotype (X for deletion) for the *Lr34a*allele fits well with genotypes of several hundred wheat accessions collected worldwide (Lagudah 2011).

The *Lr34a* allele also lost function due to a new mutation in winter wheat cultivars such as Jagger (Cao et al. 2010; Lagudah et al. 2009; Lagudah 2011). *Lr34a* is believed to confer more resistance in spring wheat from which it was initially cloned (Krattinger et al. 2009). When a population of RILs from two winter bread wheat cultivars ‘Jagger’ and ‘2174’ was tested in the field, a QTL was mapped in the *Lr34* region that explained 18 to 35% of the total phenotypic variation in leaf rust disease severity of field-tested adult plants for three years (Cao et al. 2010). Furthermore, the reported markers for polymorphisms in exons 11 and 12 do not apply to these two winter wheat cultivars, because both Jagger and 2174 carry the resistant haplotype at both exon 11 and exon 12 in *Lr34*. However, the complete sequence of the *Lr34* gene revealed the existence of a novel mutation in exon 22 of *Lr34* in Jagger (Lagudah et al. 2009; Cao et al. 2010). Due to the presence of the SNP in exon 22 causing a premature stop codon, the mutated Lr34 protein lacked 185 amino acids, including the majority of the second transmembrane domain, resulting in a non-functional form. The Jagger susceptibility allele is designated *Lr34d*, and the 2174 resistance allele is designated *Lr34e* (Lagudah 2011). The point mutation resulting in the non-functional *Lr34* in Jagger was detected in derivatives from Jagger but not in any of the spring wheat accessions (Cao et al. 2010; Lagudah et al. 2009; Lagudah 2011), suggesting that this mutation occurred during the domestication of common wheat cultivars.

A genotype carrying the susceptibility allele at any of exon 11, exon 12, or exon 22, the genotype is certainly susceptible to multiple pathogens and should not be selected. However, the previously reported molecular markers were developed on the basis of restriction enzyme digestion with PCR product, which has limited high throughput screening of alleles. In the present study, we discovered that the widely adopted winter wheat cultivar ‘Duster’ has both *Lr34a*) and *Lr34b*. The *Lr34a* and *Lr34b*genes in Duster were mapped to be tightly linked in a DH population developed from a cross of Duster with winter wheat cultivar ‘Billings’ that carries the susceptibility allele (*Lr34c*) and in an F2 population developed from a cross of Duster with 2174 that carries the resistance allele (*Lr34e*)*.* We also developed kompetitive allele-specific PCR (KASP) markers for KASP markers for allelic variation in exon 11 and exon 22 of *Lr34* that can be extensively utilized in wheat breeding programs.

## Materials and methods

### Parent description and doubled-haploid progeny evaluation

Duster (PI 644,016) is a hard red winter wheat cultivar released by the Oklahoma Agricultural Experiment Station in 2006 due to its wide adaptation across the southern Great Plains of the USA (Edwards et al. 2012). Duster traces to an F_2:3_ breeding line with pedigree W0405/NE78488//W7469C/TX81V6187. The original F_2_ population was produced in the Pioneer HRW wheat breeding program. Commercial acceptance of Duster is partly due to its consistent adult-plant resistance to leaf rust, stripe rust, and powdery mildew, and its disease-severity ratings have averaged less than 20% in breeder nurseries with natural field inoculation since 2005 in Oklahoma (Edwards et al. 2012). The resistance of Duster to multiple foliar diseases is partly due to the *Lr34* gene that is currently in low frequency among HRW wheat cultivars (Kolmer 2017; Edwards et al. 2012). Duster was also reported to have *Lr3a* and *Lr11* for seedling resistance (Kolmer 2017), as well as *Lr46* and *Lr77* for adult plant resistance (Kolmer et al. 2019). Billings (PI 656,843) is also a hard red winter wheat cultivar that was released by the Oklahoma Agricultural Experiment Station in 2009, mainly because of its excellent grain quality in combination with high yield potential (Hunger et al. 2014). Although *Lr34* is absent in Billings, it is postulated to have *Lr17* and *Lr24* (Hunger et al. 2014).

A doubled-haploid (DH) population was generated from random F_1_ plants derived from the single cross of Duster and Billings. The DH lines were genotyped using genotyping-by-sequencing (GBS) markers, and a total of 2358 GBS markers were eventually mapped in 260 DH lines (Li et al. 2015). The sequences of GBS markers on the whole genome are archived in the NCBI SRA (accession number SRP051982). The Duster x Billings DH population was used to map the *Lr34* genes in this study.

All DH lines were evaluated in the field at the Agronomy Research Station in Stillwater, OK in 2015 and 2016. The Duster × Billings DH population was arranged in the field in a replicates-in-sets design, with two replicates designed for six sets of 42 lines each but not for a seventh set of the remaining 19 DH lines. Each set also contained the two parents, and all entries were arranged within sets as a randomized complete-block design. Under natural infection and when disease incidence appeared most pronounced, each line was evaluated for reaction to leaf rust and leaf-tip necrosis. Ratings for leaf rust and leaf-tip were independently collected on 21 May 2015, and reaction to leaf rust was recorded as two infection types, resistant for uninfected and susceptible for infected leaves of adult plants. On 6 May 2016, reaction to leaf rust was recorded based on a 1-to-4 scale, 1 for resistance showing small uredinia surrounded by necrosis, 2 for moderate resistance showing moderate size uredinia surrounded by necrosis, 3 for moderate susceptibility showing moderate size uredinia surrounded by chlorosis, and 4 for susceptibility showing large uredinia without necrosis or chlorosis.

### Discovery of the heterogeneous *Lr34* genes in Duster

Previously reported three SNP markers for allelic variation in exon 11 and exon 12 (Lagudah et al. 2009) and exon 22 (Cao et al. 2010) were used to genotype Duster and Billings, while Jagger and 2174 were used as controls. In this study, a new PCR marker was developed to detect a SNP in intron 4 between the susceptible *Lr34b* gene in Duster and *Lr34c* in Billings. The primers for this marker were Lr34D-In4-F1 and Lr34D-In4-R1 (Table 1). The susceptible Lr34b gene in Duster has a ‘T’ at position 677 bp from GT at the 5′ end of intron 4, whereas Billings has an ‘A’ in the same position. The PCR products containing (TCTTC) from the susceptible *Lr34b* were digested with the restriction enzyme *Mbo* II.

**Table 1:** Primers and PCR markers used in the study

Gene/locus	Primer	Primer sequence (5′–3′)
Lr34 intron 4	Lr34D-In4-F1	ACGGCGCAATTGCCTTAATCCTC
	Lr34D-In4-R1	CACAGTGATCGCCTAGACGCC
Lr34 promoter	Lr34Prom-F2M	AAGTTCAAGGGGTTAACTACGATGAC
	Lr34Prom-R2M	CCGATTTTGATTAATCTCTAATCCCTAGTT
Lr34 exons 11–14	Cssfr6-MF2	TCTTCAAAACAGGCCAGGTTAA
	Cssfr6-MR1	TACTTTCCTGAAAATAATACAAGC
Gene expression	Lr34-E10F1	CTCATGAATTATCAAGCATGTTCAG
	Lr34-E14R1	CCAATCCAAAAGCGATATAAAATAAG
Lr34-KASP-E11	E11r-Forward	FAM-GGGAGCATTATTTTTTTCCATC***A***
	E11s-Forward	HEX-GGGAGCATTATTTTTTTCCATC***T***
	Reverse	AGCGAATCCAGTATGGAAAT
Lr34-KASP-E22	E11r-Forward	FAM-AATGTATCGTGAGAGATTTGCA***G***
	E11r-Forward	HEX-AATGTATCGTGAGAGATTTGCA***T***
	Reverse	AGGTGAATAAATATGAGCATCAGT
*TraesCS7D01G080100*	Forward	GATCTGCTGTAGAGATAGCTA
	Reverse	CACTGTCAATGAATAGACAGAAA
*TraesCS7D01G080200*	Forward	AGCGTCGTCGGCGTCTC
	Reverse	GCAGGAAAGGCTCCATGGACA
*TraesCS7D01G080300*	Forward	AAGCATCTAACATCATTGCTG
	Reverse	CCAATCCAAAAGCGATATAAAATAAG
*TraesCS7D01G080400*	Forward	CGGATCTTCCTGCAGCTA
	Reverse	GTCCTTGGTGAAGATGAATGCA
*TraesCS7D01G080500*	Forward	GATCGAGGAAGACGGC
	Reverse	AGCACTAGAGAGACCTCC

SNPs between alleles are highlighted in bold italics

In addition, a PCR marker was developed to show differences in the resistant *Lr34* gene between Duster and 2174. The first marker was involved a SNP at position 10,618 bp upstream from the start codon based on the *Lr34* gene sequence in Chinese Spring (FJ436983). Two primers, Lr34Prom-F2M and Lr34Prom-R2M (Table 1), were used to amplify a 111 bp fragment followed by digestion with the restriction enzyme *Hpa*I. After digestion, the Duster *Lr34a* showed 97 bp and 14 bp, whereas the 2174 *Lr34d* allele showed 69 bp, 28 bp, and 14 bp.

All PCRs for *Lr34* markers were performed using LongAmp *Taq* DNA polymerase (New England BioLabs) and 40 thermal cycles after denaturing at 95 °C for 5 min, with each cycle consisting of 94 °C for 30 s, 55 °C for 30 s, and 72 °C for an extension time that depended on the expected sizes of the PCR products. Unless indicated otherwise in the figure legends, the resulting PCR products were run for electrophoresis on a 1% agarose gel.

### Isolation of *Lr34-D* genes by PCR

Two primers, Cssfr6-MF2and Cssfr6-MR1 (Table 1), were designed to amplify a fragment that spanned the region from part of exon 11 to part of exon 14. The amplified PCR products were cloned into TA vectors, and four clones for each of the two alleles were sequenced.

The complete gene was isolated using two pairs of primers, ExpF1 and Cssfr6-MR1 amplifying the gene from the start codon to exon 14, and Cssfr6-MF2 and Lr34-ExpR1 amplifying the gene from exon 11 to the stop codon. The *Lr34* PCR products were cloned into TA vectors (Promega), and plasmid DNAs of several individual positive colonies were sequenced. The resistant gene and the susceptibility genes were distinguishable in the overlapped region from exon 11 to exon 14, so the complete gene for each of them was assembled. The entire sequences for each gene were aligned with published Chinese Spring genome sequences of *Lr34-A* (*TraesCS7A01G085800*), *Lr34-B* (pseudogene), and *Lr34* (*TraesCS7D01G080300*) to determine specific sequences for each of the homoeologous genes.

### *Lr34* gene expression

The total RNA was extracted from leaf samples collected from seedlings that were grown in a greenhouse with constant temperature (20–25 °C) and long day condition (16 h/8 h for light/dark). Leaf samples were also collected from the plants that were tested in a field trial at the joining stage in February and heading stage in 2012, Stillwater Research Station, Oklahoma State University. The tip and base tissues of the flag leaves from adult plants were separated to extract RNAs.

In order to test if two *Lr34* genes in Duster were expressed, two specific primers Lr34-E10F1 and Lr34-E14R1 (Table 1) were simultaneously used to amplify *Lr34* transcripts containing exon 11 and exon 12, where the resistance and susceptibility alleles can be distinguished by using the restriction enzyme *Fnu*4HI. The PCR was performed by using LongAmp *Taq* DNA polymerase (New England BioLabs) and 35 thermal cycles after denaturing at 95 °C for 5 min, with each cycle consisting of 94 °C for 30 s, 55 °C for 30 s, and 72 °C for 90 s. Lr34ExpF1 and Lr34ExpR1 were paired to amplify *Lr34* transcripts.

### Chromosomal fragment duplication by copy number of genes.

*Lr34* was cloned by using a physical contig of BAC clones (GenBank accession number: FJ436983) (Krattinger et al. 2009). The corresponding genes in the physical contig have been annotated in the wheat genome sequence at IWGSC RefSeq v1.0 (https://urgi.versailles.inra.fr/blast/?dbgroup=wheat_iwgsc_refseq_v1_chromosomes), where *Lr34* is annotated as *TraesCS7D01G080300.* Specific primers (Table 1) were designed for five genes, including *Lr34*, two neighboring genes on its distal side (*TraesCS7D01G080100* and *TraesCS7D01G080200*) and two neighboring genes on its proximal side(*TraesCS7D01G080400* and *TraesCS7D01G080500*). Quantitative RT-PCR (qRT-PCR) was used to determine copy number of each gene by the SYBR Green PCR Master Mix, and *TaCO2* was used as an endogenous control*.* Sequence information for these primers is provided in Table 1, and primers for *TaCO2* control were cited from previous studies (Díaz et al. 2012; Li et al. 2015). Genome DNAs of Duster and Billings were used as PCR template. The qRT-PCRs were carried out on a CFX96™ Real-Time system (Bio-Rad laboratories, Hercules, CA) using SsoAdvanced Universal SYBR Green Supermix (Bio-Rad laboratories, Hercules, CA). Assays were tested in 96-well formats and set up as 10 μl reactions (3 μl gDNA at 35 ng/μl, 5 μl of SsoAdvanced Universal SYBR Green Supermix, 0.3 μl of 10 mM forward primer, 0.3 μl of 10 mM reverse primer, and 1.4 μl H_2_O). PCR program was: denatured at 95 °C for 3 min, with 39 cycles (95 °C for 30 s; 57 °C for 30 s; 72 °C for 30 s) for melt curve analysis from 65 °C to 95 °C with 0.5 °C increments for 5 s per step. Six technical repeats were performed for each sample.

### Development of KASP-based assays for multiple alleles of *Lr34*

Four variation sites in intron 4, exon 11, exon 12, and exon 22 have been reported (Krattinger et al. 2009; Lagudah et al. 2009; Cao et al. 2010). KASP markers were tested for each of these variation sites, but only two KASP markers were finally developed for *Lr34*: one for allelic variation in exon 11 and the other for allelic variation in exon 22.

The Duster × Billings and Duster × 2174F_2_ lines were developed for mapping of the heterogeneous *Lr34* in Duster. DNAs of heterozygous alleles for *Lr34* in the F_2_ lines of Duster × Billings and Duster × 2174 developed in this study and Jagger × 2174 used in the previous study (Cao et al. 2010) were also used to validate the KASP markers.

KASP primers were designed following standard KASP guidelines (LGC Genomics, Hoddesdon, UK), and the primer sequences are provided in Table 1. The allele-specific primers were designed carrying the standard FAM (5′-GAAGGTGACCAAGTTCATGCT-3′) and HEX (5′-GAAGGTCGGAGTCAACGGATT-3′) tails and with the targeted SNP at the 3′ end. A common genome specific primer was designed, and the total amplicon length was 174 bp for Lr34-E11-KASP and 130 bp for Lr34-E22-KASP excluding FAM and HEX. The primer mixture comprised 46 μl ddH2O, 30 μl common primer (100 μM), and 12 μl of each tailed primer (100 μM). Assays were tested in 96-well formats and set up as around 10 μl reactions (4.83 ul DNA at 40 ng/μl, 5 μl of 1 × KASP master mixture, and 0.14 μl of primer mixture).

For Lr34-E11-KASP, PCR cycling was performed using the following protocol: hot start at 94 °C for 15 min, followed by ten touchdown cycles (94 °C for 20 s; touchdown at 61 °C initially and decreasing by − 0.6 °C per cycle for 60 s), followed by 45 additional cycles (94 °C for 20 s; 55 °C for 60 s). For Lr34-E22-KASP, PCR was performed in the same program as did for the InDel in exon 11 of *Lr34*, except 30 additional cycles at the last step. Three replicates for each genotype were performed.

## Results

### Discovery of two *Lr34* genes in Duster

**Fig. 1:**
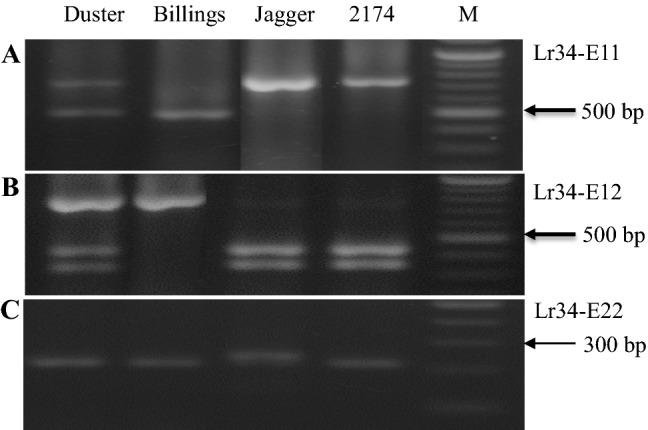
Heterogeneous *Lr34-D* genes in Duster. (**A**) PCR marker for exon 11. (**B**) PCR marker for exon 12. (**C**) PCR marker for exon 22. M: indicates a DNA marker. Duster in the first lane shows the presence of heterogeneous *Lr34*genes for all of the three markers. Billings shows a susceptibility allele at exon 11 and exon 12, Jagger shows a susceptibility allele at exon 22, and 2174 shows a resistant allele at each of the three markers. M indicates marker for 100 bp ladder

Three published markers for variation in exons 11, 12, and 22 of *Lr34* are routinely used to genotype germplasm in the Oklahoma State University wheat breeding program. Surprisingly but consistently, when the marker for allelic variation in exon 11 was used to genotype, Duster showed both the larger DNA fragment representing the *Lr34a*allele for resistance and the smaller DNA fragment representing the *Lr34b* allele for susceptibility (Fig. 1A). Similarly, when the marker for allelic variation in exon 12 was tested, Duster showed both the double smaller DNA fragments representing the *Lr34a* allele for resistance and the larger DNA fragment representing the *Lr34b*allele for susceptibility (Fig. 1B). When the PCR marker for the polymorphism in exon 22 was tested, Duster showed the*Lr34* allele for resistance only (Fig. 1C). All other cultivars, including Jagger, 2174, and Billings, tested by the three *Lr34* markers showed the same homozygosity and homogeneity as expected. As shown in Fig. 1A and B), the DNA fragments for the resistance allele and the susceptibility allele of *Lr34* had similar intensity, suggesting the heterozygous pattern of the *Lr34* gene in Duster. Genotyping of DNAs from individual plants of Duster, even from different seed sources, produced consistent results for the *Lr34* markers, supporting the notion that the observed heterozygosity in Duster was not caused by genetic background impurity.

In order to exclude any possibility that the heterogeneous PCR products were not from *Lr34* but from homoeologous *Lr34-A* or *Lr34-B* gene in Duster, primers Cssfr6-MF2 and Cssfr6-MR1 were used to amplify a *Lr34* fragment that covered both exon 11 and exon 12 from Duster, and four clones were sequenced. Two clones representing *Lr34-Da* showed the same sequence as the XXX-T haplotype (X for deletion) for the *Lr34a*allele, whereas two clones representing *Lr34b* showed the same sequence as the TTC-C haplotype for the *Lr34b* allele. These results supported that Duster had two copies of *Lr34.* The two *Lr34* genes in Duster were temporarily designated *Lr34a*for the resistance gene and *Lr34b* for the susceptibility gene*.* It was intriguing to know where *Lr34a* and *Lr34b* are located in the Duster genome and why Duster had heterogeneous *Lr34* genes.

### Genetic mapping of two *Lr34* genes

The most convincing evidence for the existence of the *Lr34a* and *Lr34b1* genes in Duster was that the presence or absence of each of the *Lr34a* and *Lr34b* genes was mapped in the Duster × Billings population consisting of 260 DH lines. The DH population was generated using Duster to cross with Billings (Li et al. 2015)*.* Duster carries a resistance gene *Lr34a*, which was confirmed in this study (Fig. 1A–C), whereas Billings carries a *Lr34* gene for susceptibility (Hunger et al. 2014). Two PCR markers were used to map *Lr34* in the DH population.

**Fig. 2:**
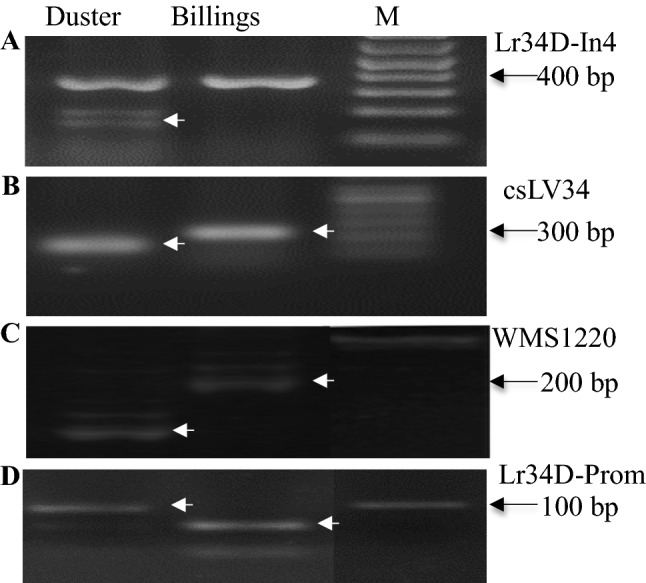
PCR markers for *Lr34a* and *Lr34b* in Duster. (**A**) PCR marker for a SNP in intron 4. The *Mbo* II-digested PCR products showed 360 bp and 157 bp-203 bp for the Duster *Lr34b* but only 360 bp for the Billings*Lr34a*. (**B**) csLV34 marker. (**C**)WMS1220 marker. (**a**–**c**) were for allelic variation between Duster and Billings. (**D**) PCR marker for a SNP in the promoter region of *Lr34* between Duster *Lr34a* and 2174 *Lr34c*. The *Hpa*I-digested PCR products showed 97 bp and 69 bp for the Duster allele but 69 bp for the 2174 allele. In addition, 14 bp and 28 bp are in both of the alleles. M indicates marker for 100 bp ladder. Arrows point to DNA fragments that are mapped

The first PCR marker was used to map the *Lr34a* gene. As shown in Fig. 1A, Lr34-E11 is a dominant marker (the upper band on Duster, Jagger and 2174) that was present in Duster but absent in Billings*.* This marker was used to genotype the 260 DH lines, 129 lines were found to have the *Lr34a* gene, and the remaining 131 lines did not have this gene. The second marker is a PCR marker that was developed based on a SNP in intron 4 to map the *Lr34b*gene. This SNP was mapped using primers Lr34-D-In4-F1 and Lr34-D-In4-R1 to amplify a fragment, which was digested with the restriction enzyme *Mbo* II, resulting in polymorphic DNA fragments (Fig. 2A). Duster has both resistance allele (the undigested upper band) and susceptibility allele (the two digested lower bands), whereas Billings has the resistance allele (Fig. 2A). Overall, the susceptible intron 4 in Duster was tightly linked with the *Lr34a* gene that was mapped in the 129 lines using the first marker Lr34-E11. These results indicated that Duster has both the resistance and susceptibility alleles at both intron 4 and exon 11, while Billings has the resistance allele in intron 4 but the susceptibility allele in exon 11. The two genes in Duster are tightly linked in the 260 DH lines of Duster × Billings.

**Fig. 3:**
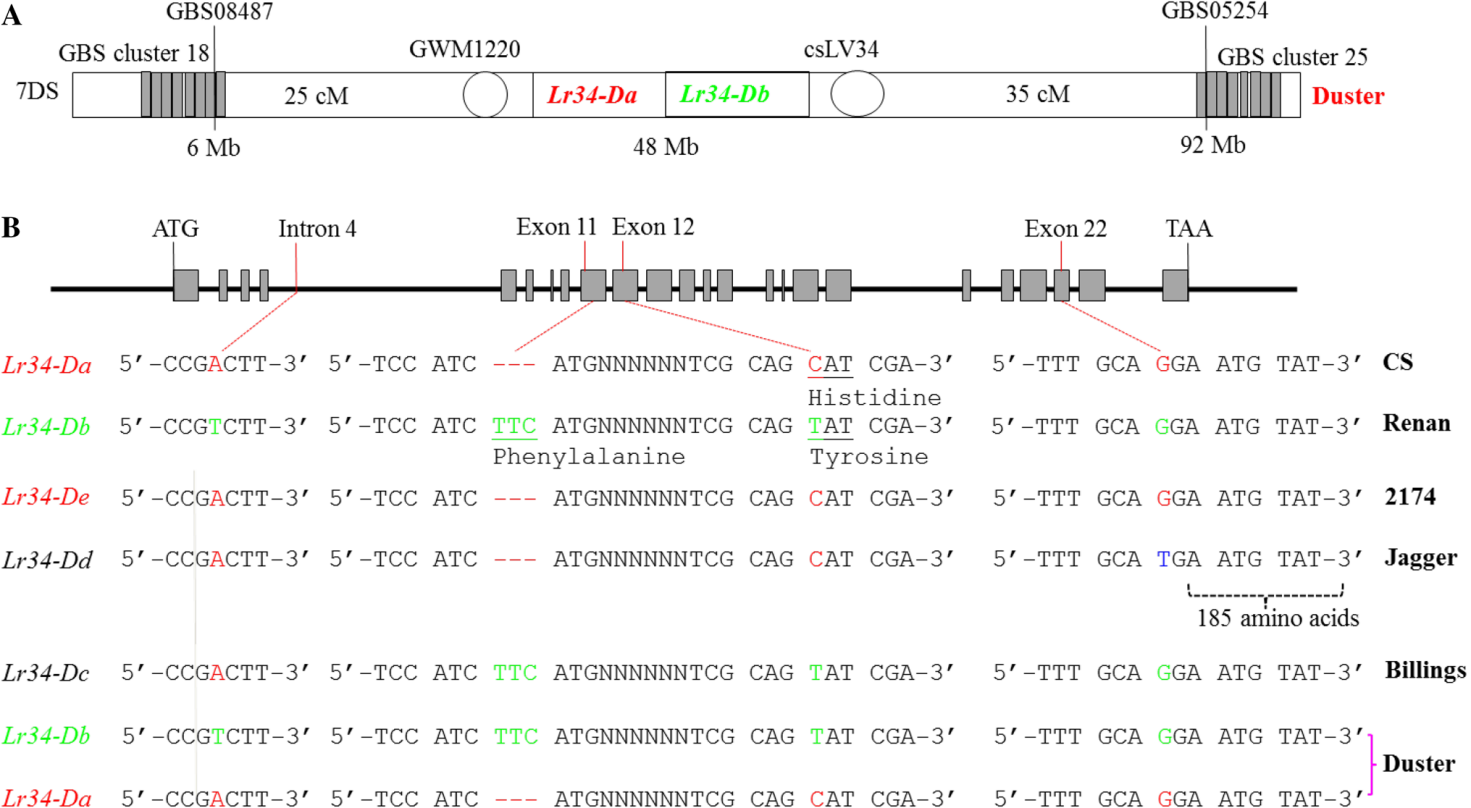
Diagram of haplotypes at *Lr34*. (**A**) Location of the Duster *Lr34a* and *Lr34b* genes on chromosome 7D. The two genes are linked with GWM1220 and csLV34 but separated from the two GBS markers clusters (18 and 25). The physical location of two GBS markers, GBS08487 and GBS05254, is indicated. (**B**) Comparison between different *Lr34*alleles. The sequences around four reported polymorphic sites in intron 4, exon 11, exon 12, and exon 22 are provided. The names of the alleles are cited (Lagudah 2011). The position of the premature stop codon TGA resulting in a lack of 185 amino acids in Jagger is indicated

The sequence of the *Lr34* gene was used to search in the recently released IWGS databases of Chinese Spring genomic sequences, IWGSC RefSeq v1.0 (IWGSC 2018), allowing the determination of this gene position from 48,964,777 bp to 48,952,973 chromosome 7DS. A total of 2358 GBS markers were mapped in the Duster × Billings population of 260 DH lines, including two unlinked clusters of GBS markers, cluster 18 (28 markers) and cluster 25 (16 markers) that were assembled and mapped on chromosome 7D (Fig. 3A). Sequences of the GBS markers were used to search in the IWGS databases, allowing the determination of cluster 18 on chromosome 7DS and cluster 25 on chromosome 7DL. For example, GBS08487 included in cluster 18 was found to have 100% identity to CS sequence, and it is a single copy located from 6,026,380 bp to 6,026,442 bp on chromosome 7D. GBS05254 included in cluster 25 was found to have 100% identity to CS sequence, and it is a single copy located from 91,742,616 bp to 91,742,679 bp on chromosome 7D (Fig. 3A). However, *Lr34* was mapped 25 cM to cluster 18 and 35 cM to cluster 25 (Fig. 3A). These results suggested that the linked *Lr34*genes in an approximate 85 Mb region where no GBS markers were observed.

In order to ensure that the *Lr34* genes are indeed located on chromosome 7DS, several markers that are reported to reside at the *Lr34* locus were screened for polymorphisms between Duster and Billings. Two markers were polymorphic, one for *csLV34* (Fig. 2B), which is a STS marker linked with *Lr34* (Lagudah et al. 2006), and the other for *Xgwm1220* (Fig. 2C), which is a SSR marker linked with *Lr34* (Lillemo et al. 2013). Among the first 96 DH lines, crossovers were found between *csLV34* and *Lr34a*in two DH lines (DH15 and DH39) and no crossover was found between *Xgwm1220* and *Lr34a*, further confirming that the duplicated *Lr34a* and *Lr34b* genes were located on chromosome 7DS in Duster.

The genomic sequences for each of *Lr34a* and *Lr34b* were determined by using primers to amplify two overlapping fragments from each gene. The first fragment extending from the start codon to exon 16 was amplified using primers Lr34D-ExpF1 and Cssfr6-MR1. The second fragment extending from exon 11 to the stop codon was amplified using primers Cssfr6-MF2 and Lr34D-ExpR1. As shown in Fig. 3B, sequencing of the complete genes from start codon to stop codon for translation showed that Duster had both *Lr34a* of *In4r-E11r-E12r-E22r* and *Lr34b* of *In4s-E11s-E12s-E22s* in the same sequences as previously reported (Krattinger et al. 2009; Lagudah et al. 2009)*. Lr34a* was indicative of the *In4r*-*E11r-E12r-E22r* allele for resistance, and *Lr34b* was indicative of the*In4s*-*E11s-E12s-E22r* allele for susceptibility. Billings had the same allele as *Lr34c*, which is the structure of *In4r*-*E11s-E12s-E22r* (Lagudah et al. 2009). The haplotype of *Lr34*in Duster was hereafter referred to as the *Lr34ab* allele for its linked *Lr34a*/*Lr34b* genes.

### Allelic variation between *Lr34a* and *Lr34c*

Since the *Lr34a* in Duster and *Lr34c* in 2174 have identical sequences from the start codon to the stop codon for translation (Fig. 3B), all of the *Lr34* markers produced the same pattern for *Lr34a* in Duster and *Lr34c* in 2174. More regions at the promoter and the 3′ end were sequenced, and PCR markers were developed to distinguish*Lr34a* in Duster and *Lr34c* in 2174. Approximately 10 kb for the upstream of the 5′ end and downstream of the 3′ end of *Lr34* was cloned and sequenced for each of Duster and 2174. A SNP in the upstream at position 10,540 bp from the start codon was found between *Lr34a* in Duster and *Lr34e*in 2174, and a PCR marker for this SNP was developed using primers Lr34Prom-F2M and Lr34Prom-R2M (Fig. 2D). Among 196 F_2_ lines of Duster × 2174, both *Lr34a* and *Lr34b* in Duster were mapped to be allelic to *Lr34e* in 2174.

### Functional characterization of *Lr34ab* in Duster

**Fig. 4:**
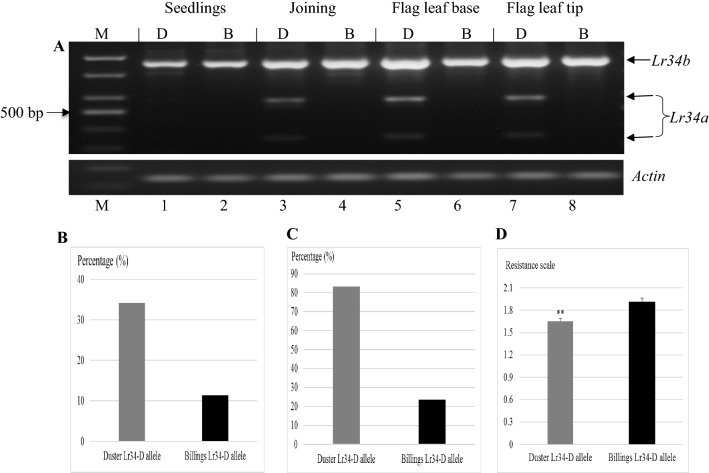
Comparison of *Lr34* function between Duster and Billings. (**A**) Expression of *Lr34* at different stages. Duster (D) and Billings (B) at seedlings, leaves at joining stage, flag leaf base, flag leaf tip. Billings has *Lr34c* only, whereas Duster has *Lr34a* and *Lr34d* that was digested with *Fnu*4HI. Actin was used an endogenous gene for control. (**B**) Duster allele versus the Billings allele in reaction to leaf rust. Values for *Y* axis are percentage of the resistance lines to the total DH lines carrying the Duster *Lr34* allele or the Billings *Lr34* allele. (**C**) Duster allele versus to Billings allele in tip necrosis. Values for *Y* axis are numbers of plants; *Y* is percentage of the lines with tip necrosis to the total DH lines carrying the Duster *Lr34* allele or the Billings *Lr34* allele. (**D**) Comparison of the Duster allele versus the Billings allele in reaction to leaf rust, rated on, 6 May 2016, using a 1-to-4 scale. Values for *Y* axis are the average ratings of DH lines that have the same *Lr34* allele to leaf rust, *n* = 138 for the Duster allele and *n* = 124 for the Billings allele. The bar indicates standard error; asterisk indicates that the difference is highly significant (*p* < 0.0001)

In order to determine if either or both of the *Lr34a* and *Lr34b* genes in Duster were expressed, the *Lr34* genes were tested at the transcriptional level in leaf samples collected at different stages. As shown in Fig. 4A, *Lr34c* in Billings and *Lr34b* in Duster (the larger DNA fragment) showed similar expression patterns at different stages. Duster showed expression of additional *Lr34a*gene (the middle and smaller DNA fragments) that was distinguished by digestion of the same cDNA samples containing *Lr34b* with a restriction enzyme *Fnu*4HI (Fig. 4A). Sequencing cDNA clones from Duster also confirmed the presence of both *Lr34a* and *Lr34b* in the Duster RNA samples.

Compared with the Billings *Lr34c* allele, the Duster *Lr34ab* allele showed increased protection against leaf rust, when the population of Duster × Billings DH lines was tested in the field in 2015. In 114 DH lines carrying the Duster *Lr34ab* allele, 34.2% of them had no leaf rust pustules and 65.8% of them were infected. Among 114 DH lines carrying the Billings *Lr34c* allele, only 11.4% of them had no leaf rust pustules and 88.6% of them were infected (Fig. 4B). The appearance of leaf-tip necrosis, indicative of *Lr34*, also showed significant difference between the Duster allele and the Billings allele; and up to 83.4% of the 114 DH lines carrying the Duster *Lr34ab* allele showed the appearance of leaf-tip necrosis (Fig. 4C). When the population of Duster × Billings DH lines was tested in the field in 2016, the DH lines carrying the Duster *Lr34ab* allele and the DH lines carrying the Billings *Lr34c* gene also showed significant difference in response to leaf rust at the adult plants. Respective mean ratings for the two RILs groups were 1.65 for the Duster allele and 1.9 for the Billings allele (*p* < 0.0001), indicating that the Duster allele had increased protection against leaf rust (Fig. 4D).

### Duplication of a chromosomal fragment covering *Lr34*

**Fig. 5:**
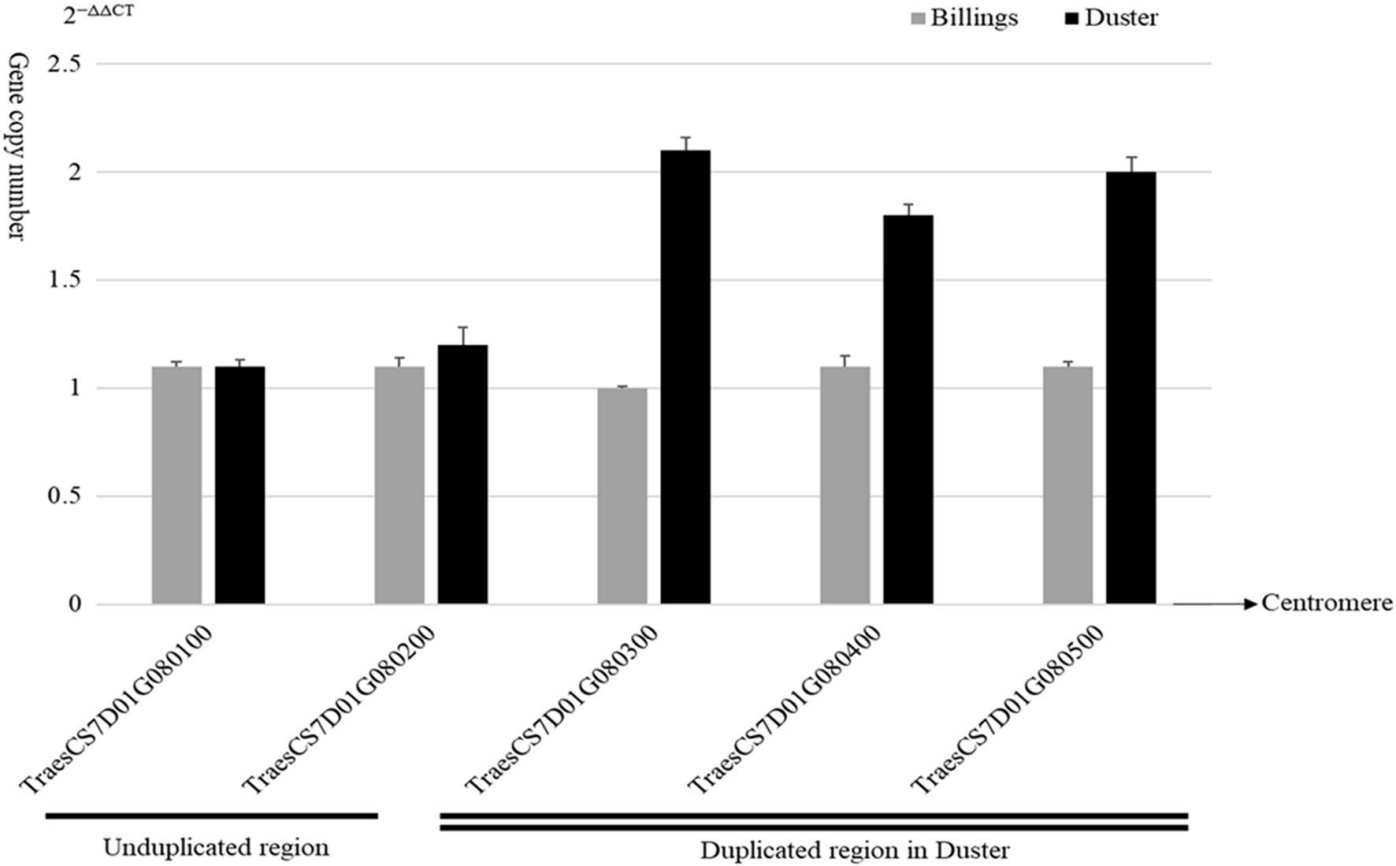
A chromosomal fragment duplication event in Duster but not in Billings. qRT-PCRs were used to determine copy number of five genes including *Lr34*, two neighboring genes on its distal side (*TraesCS7D01G080100* and *TraesCS7D01G080200*) and two neighboring genes on its proximal side (*TraesCS7D01G080400* and *TraesCS7D01G080500*). The centromere is pointed out with an arrow. Genome DNAs of Duster and Billings were used as qRT-PCR template. *TaCO2* was used as an endogenous control*.* Copy number is shown using the values calculated by the 2^(−ΔΔCT)^ method, where CT is the threshold cycle. Bar indicates standard error. The single line under *TraesCS7D01G080100* and *TraesCS7D01G080200* indicates that the two genes have one copy in both Billings and Duster. The double lines under *TraesCS7D01G080300*, *TraesCS7D01G080400*, and *TraesCS7D01G080200* indicate that each of these three genes has two copies in Duster (black graph) but one copy in Billings (gray graph)

In order to determine if a single gene *Lr34* or a chromosomal region containing in *Lr34* Duster was duplicated, a qRT-PCR method was used to successfully determine copy number of genes in the chromosomal region containing *Lr34.* As shown in Fig. 5, *Lr34* and all four neighboring genes in Billings showed one copy. In Duster, *Lr34* and the two genes on its proximal side to the centromere showed two copies, but the two genes on the distal side of *Lr34* showed only one copy. This result indicated that the chromosomal fragment duplication including *Lr34* and at least the two neighboring genes on its proximal side occurred in Duster but not in Billings.

### Development of KASP-based assays for multiple alleles/haplotypes of *Lr34*

**Fig. 6:**
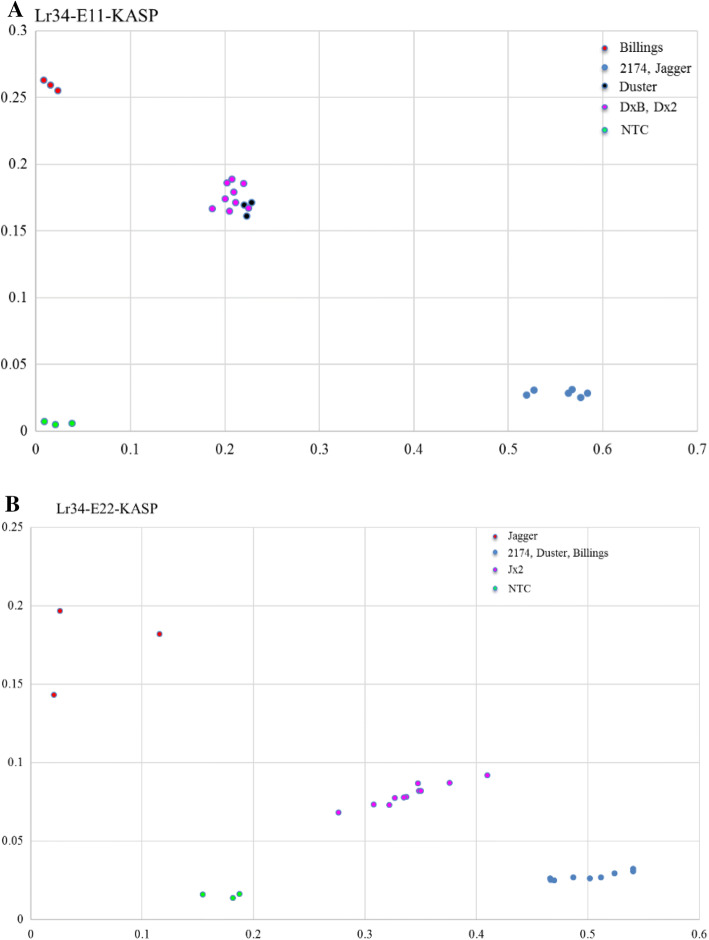
KASP markers for Lr34. (A) Lr34-E11-KASP for allelic variation in exon 11. (B) Lr34-E22-KASP for allelic variation in exon 22. DxB is DNA from the heterozygous allele of Duster and Billings that was confirmed using the PCR marker for allelic variation in exon 11 of *Lr34*. Dx2 is DNA from the heterozygous allele of Duster and 2174 that was confirmed using the PCR marker for allelic variation in exon 11 of *Lr34*. Jx2 is DNA from the heterozygous allele of Jagger and 2174 that was confirmed using the PCR marker for allelic variation in exon 22 of *Lr34*. Scatter dots with different colors show clustering of genotypes on the *X*- (FAM) and *Y*- (HEX) axes. Green dots represent the NTC (non-template control)

Two KASP markers, Lr34E11-KASP and Lr34-E22-KASP, were developed to distinguish multiple alleles of *Lr34* in wheat. The forward primer for Lr34-E11-KASP has specific nucleotide at its 3′ end, ‘T’ that is the first nucleotide of ‘TTC’ encoding phenylalanine present in exon 11 in the susceptibility allele (E11s) but ‘A’ that is located after the deleted ‘TTC’ in the resistance allele (E11r). As shown in Fig. 6A, Lr34-E11-KASP was used to effectively distinguish the susceptibility allele being in Billings and the resistance allele being in Jagger and 2174. Duster, carrying heterogeneous E11r and E11s, showed the same pattern as the heterozygous alleles of Duster (E11r and E11s) × Billings (E11s only), indicating that one copy *Lr34-E11s* in Duster was not distinguishable from two *Lr34-E11s* copies in the heterozygous Duster × Billings allele at *Lr34*. Duster also showed the same pattern as the heterozygous alleles of Duster (E11r and E11s) × 2174(E11r only), indicating that one copy *Lr34-E11r* in Duster was not distinguishable from two *Lr34-E11r* copies in the heterozygous Duster × 2174 allele at *Lr34*.

The forward primer for Lr34-E22-KASP has a specific nucleotide at its 3′ end, ‘T’ that is the first nucleotide of ‘TGA’ encoding a stop codon present in exon 22 in the susceptibility allele (E22s) but ‘G’ in the same position for the resistance allele (E22r). As shown in Fig. 6B, Lr34-E22-KASP was used to effectively distinguish the resistance allele present in 2174, Duster and Billings and the susceptibility allele present in Jagger. Lr34-E22-KASP showed a single allele in exon 22 in Duster, compared with the heterozygous allele in Jagger × 2174.

The Duster *Lr34ab* allele was surveyed for its frequency among parental lines used for the US T-CAP populations, recently released wheat cultivars in the southern Great Plains and relevant germplasm, and current OSU breeding lines (Fang et al. 2011). Except Duster, no other cultivar tested was found to have *Lr34ab*.

## Discussion

*Lr34* has been extensively studied as a single gene since it was reported. In this study, we discovered that Duster had the new resistance allele *Lr34ab* allele containing both *Lr34a *and *Lr34b* genes, which are duplicated or heterogeneous. In addition to genetic confirmation of seed purity, a DH population and an F_2_ population were used to map the two *Lr34* genes in Duster. PCR markers for presence or absence of each of *Lr34a *and *Lr34b* genes in Duster were mapped in the two independent populations, providing more convincing evidence for the existence of the *Lr34a *and *Lr34b* genes in a single genome in Duster. *Lr34a* in Duster has the *In4r-E11r-E12r-E22r* exon structure that is the same as CS, and *Lr34b* in Duster has *In4s-E11s-E12s-E22r* structure that is the same as Renan. Therefore, the haplotype of *Lr34ab* is novel.

A conventional approach to confirm the existence of duplicated DNA fragments or genes in a genome is to use P^32^-labled DNA probe or fluorescence in situ hybridization (FISH) to hybridize with a Southern blot of total gDNAs of the genome, and a conclusion is drawn based on the DNA band pattern and intensity of digested DNAs on the blot (Yan et al. 2004). A qRT-PCR method is also used to successfully determine copy number of genes in previous studies (Diaz et al 2012; Li et al. 2015). Since the wheat genome has been sequenced and the genes in a specific genome region are ordered, the qRT-PCR method can be used to determine if a single gene is duplicated or a genomic region containing the ordered genes is duplicated. The qRT-PCR method is relatively easier and also provides convincing results compared with the conventional FISH method. We used the qRT-PCR method to determine copy number of neighboring genes adjacent to *Lr34* in the reported BAC clone. The order of genes in the BAC clone containing *Lr34* (Krattinger et al. 2009) is the same as annotated in the wheat genome sequence at IWGSC RefSeq v1.0. We concluded that the chromosomal fragment including *Lr34* and at least two neighboring genes on its proximal side but excluding genes on its distal side occurred was duplicated in Duster.

It is not yet known, however, how the chromosomal fragment duplication occurred. It is significant that the *Lr34-B* gene on homoeologous chromosome 7B was translocated into homoeologous chromosome 4A (Krattinger et al. 2011); therefore, three homoeologous *Lr34* genes are located on homoeologous chromosomes 7D, 7A, and 4A. It was reported that there is clear chromosome distortion on the short arm of chromosome 7DS (Wang et al. 2009). It is possible that the chromosome structures in the region covering *Lr34* of homoeologous chromosome 7 cause the translocation and the duplication. In the wheat literature, translocation events of 7B/4A and 4A/5A have been known for many years, but the mechanisms underlying the translocations are not yet known. Much more work is required to unravel the mystery.

It is also intriguing why the chromosomal fragment occurred in Duster, a unique winter wheat cultivar. It is also found that that Duster has the *QYld. osu-1BS* allele on chromosome arm 1BS that confers higher grain yield, which is a unique haplotype in the whole exome capture dataset, compared with 57 cultivars and breeding lines with various genetic backgrounds (Kan et al. 2020). It is not yet known where those unique sequences in Duster originated.

It was not surprising that the Duster *Lr34ab* haplotype was effective against leaf rust, since it has *Lr34a* that has identical sequence to *Lr34e* in 2174. *Lr34e* in 2174 was previously demonstrated to be effective when tested in Oklahoma (Cao et al. 2010). The linkage of *Lr34ab* genes in Duster with tip necrosis further supported the existence of the functional *Lr34a* in Duster. Even if a cultivar has a functional *Lr34* gene, most of its transcripts were not completely spliced in a correct pattern, resulting in partial resistance of this gene (Fang et al. 2017). Therefore, the resistance of the *Lr34* gene against leaf rust could be increased by eliminating or mutating regulators that cause mis-splicing events in wheat (Fang et al. 2017). Previous transformation studies on *Lr34a* showed that when the resistance gene *Lr34a* was transferred to a cultivar carrying the *Lr34b* gene, the transgenic wheat showed sufficient resistance to leaf rust compared to natural wheat containing an endogenous *Lr34a* gene, demonstrating function of *Lr34a* in wheat (Risk et al. 2012; Rinaldo et al. 2017), barley (Boni et al. 2018), rice (Krattinger et al. 2016), Sorghum (Schnippenkoetter et al. 2017), and maize (Sucher et al. 2017). Transgenic wheat, however, has not yet been approved for commercial use. So far, the deployment of the *Lr34a* gene is still based on the conventional breeding approach, by which a resistant allele from a donor is introduced to a recipient cultivar carrying a susceptibility allele; therefore, only a single copy of the resistant *Lr34a* allele is yet achieved in a cultivar. The co-existence of *Lr34a* and *Lr34b* at the same locus in Duster provides an intriguing possibility that two natural resistance genes could be combined into a single genotype by conventional crossing methods, one from *Lr34a* in Duster and the other from 2174 or its descendent that carries the resistant gene *Lr34e*. The CNV resulting from the duplication of a gene is difficult to work with, because the native gene and the duplicated gene(s) are very similar in sequence (Dubcovsky and Dvorak 2007; Saintenac et al. 2011). It is even much more difficult to work with duplicated genes in bread wheat, as this species already has three homoeologous genes that have similar sequences.

Duster and its derivative have been elite genetic source in breeding programs of winter wheat in Oklahoma and surrounding states. Previous molecular markers for *Lr34* were developed based on restriction enzyme digestion for detection of allelic variation in this gene. We attempted to develop KASP marker for each allelic variation intron 4, exon 11, exon 12, and exon 22, but the KASP system did not work for intron 4 or exon 12. Intron 4 is not involved in the function of Lr34, and exon 12 is always linked with exon 11 in their genotypes. The development of KASP markers for SNPs/InDels at exon 11, as well as exon 22 of *Lr34* would accelerate identification and deployment of the resistance alleles in wheat breeding.
